# Common Chiffchaffs (*Phylloscopus collybita*) Diverge in a Genomic Region Associated With Migration Differences in Willow Warblers (*Phylloscopus trochilus*)

**DOI:** 10.1111/mec.70462

**Published:** 2026-07-09

**Authors:** Violeta Caballero‐Lopez, Alexander Mackintosh, Diana Ekman, Estelle Proux‐Wéra, Max Lundberg, Gintaras Malmiga, Daria Shipilina, Michal Polakowski, Michaëla Berdougo, Łukasz Jankowiak, Staffan Bensch

**Affiliations:** ^1^ Department of Biology Lund University Lund Sweden; ^2^ Department of Ecology and Genetics, Evolutionary Biology Centre Uppsala University Uppsala Sweden; ^3^ Department of Biochemistry and Biophysics, National Bioinformatics Infrastructure Sweden Science for Life Laboratory Stockholm University Solna Sweden; ^4^ State Scientific Research Institute Nature Research Centre Vilnius Lithuania; ^5^ Department of Ecology and Genetics, Program of Evolutionary Biology Uppsala University Uppsala Sweden; ^6^ Department of Ecology and Anthropology, Institute of Biology University of Szczecin Szczecin Poland

**Keywords:** divergence, geolocators, migration, migratory direction, migratory divide

## Abstract

Despite technological advances in both tracking and sequencing technologies, finding the genetic mechanisms behind migratory traits remains a challenge. Recent studies have shown that migratory direction in European willow warblers ( *Phylloscopus trochilus* ) is mainly influenced by a repeat‐rich region named MARB. Whether this genetic basis for orientation is unique to the willow warbler or instead shared by other closely related species is currently unknown. Therefore, here we investigate a close relative, the common chiffchaff ( *Phylloscopus collybita* ) by combining whole‐genome resequencing data and geolocator tracks from two subspecies, *P. c. collybita* and *P. c. abietinus*. As in willow warblers, our geolocator data reveals that the two chiffchaff subspecies differ in orientation, with westward and eastward migratory directions from their breeding grounds in Sweden and south of the Baltics. Genome‐wide divergence between the subspecies is low and consistent with a recent split within the last 100,000 years, as provided by demographic analyses. The divergence between subspecies is likely a result of shifts in allele frequencies across many loci although signals of differentiation are concentrated in the Z chromosome. Most differences, however, are found in the MARB region, raising the possibility that MARB contributes to orientation in chiffchaffs and may have evolved in parallel to willow warblers. Given the repeat‐rich structure of this region, its characterization using short‐read NGS sequencing remains challenging, and the diversity and nature of MARB haplotypes must be interpreted with caution.

## 1 Introduction

Evidence from solitary migrants shows that avian migratory behaviour has a strong genetic foundation (Berthold et al. 1990; Helbig 1991). A large part of the field of bird migration has therefore focused on finding the genetic mechanisms behind this fascinating phenomenon. With the rapid development of affordable and sophisticated sequencing technologies, combined with tracking devices, several studies aimed at finding the exact genes behind migratory traits through comparative genomics (Lundberg et al. 2013; Zhang et al. 2014; Delmore et al. 2016). However, studies highlight different genetic regions that correlate with different traits of the migratory syndrome. For instance, different populations of Swainson's thrushes ( *Catharus ustulatus* ) differ in ‘migratory sleeplessness’ which is associated with the *CPNE4* gene (Ruegg et al. 2014), while a study in *Vermivora* warblers shows a strong association between the *VPS13A* gene and wintering longitude (Toews et al. 2019). In blackcaps ( *Sylvia atricapilla* ), SNPs in non‐coding regions have been linked to the transition to residency (Delmore et al. 2020). The recent literature emphasizes this diversity, proposing that migratory phenotypes across species could arise from different genetic mechanisms (Delmore et al. 2020; Caballero‐Lopez and Bensch 2024; Zamudio‐Beltrán et al. 2025) as opposed to a common basis for all. This aligns with the notion that the migratory behaviour is not a monophyletic trait and has instead evolved several times across the bird phylogeny (Alerstam and Hedenstrom 1998; Zink 2011). However, because most studies compare distantly related species, it remains unclear how repeatable genotype–phenotype associations are within closely related lineages. Our system provides a unique opportunity to address this question by examining different species within the *Phylloscopus* genus.

A Palearctic songbird species, the willow warbler ( *Phylloscopus trochilus* ) has been the subject of several migration studies as it is a strict long‐distance migrant with two distinct migratory phenotypes in Europe (Bensch et al. 1999; Bensch, Åkesson, and Irwin 2002; Lundberg et al. 2013, 2017). *P. t. trochilus* breeds across West and Central Europe and migrates SW to its wintering grounds in western Africa whereas *P. t. acredula* has a more northern breeding distribution and migrates SE to eastern and southern Africa (Hedenström and Petterson 1987). Both subspecies come into contact in their breeding ranges in what is described as narrow ‘migratory divides’ in central Scandinavia and east of the Baltic Sea (Bensch et al. 1999, 2009). The integrity of these divides, combined with documented random mating (Liedvogel et al. 2014) suggests that selection is acting against hybrids, possibly due to maladaptive migratory routes (Zhao et al. 2020). Both subspecies present slight plumage and biometric differences that overlap greatly (Bensch et al. 2009; Liedvogel et al. 2014). Their genomes are virtually undifferentiated except for four regions encompassing three inversion polymorphisms in chromosomes 1, 3, and 5 that date back to approximately 1.2 My ago (Lundberg et al. 2017, 2023), and an independent repeat‐rich region known as the Migration‐Associated Repeat Block (MARB) that has not yet been assigned to a specific chromosome (Caballero‐López et al. 2022; Sokolovskis et al. 2023). Three of these regions are directly or indirectly associated with migration differences (Lundberg et al. 2017; Sokolovskis et al. 2023) whereas the inversion polymorphism in chromosome 3 is associated with a climate gradient (elevation and latitude) in the breeding range (Larson et al. 2014).

The common chiffchaff ( *Phylloscopus collybita* ) is the closest relative to the willow warbler with an estimated time of divergence of 5 My (Alström et al. 2018). It is recognized as an incipient species complex, containing eight taxa with species or subspecies status (Clement and Helbig 1998; Alström et al. 2018; Rheindt et al. 2025). In Sweden, the *abietinus* subspecies historically occupies the central and northern regions, whereas *collybita* is a more recent colonizer that has entered Sweden from the south and is rapidly moving north (Figure S1), where contact and potentially a hybrid zone is starting to form (Hansson et al. 2000). However, the density of chiffchaffs in the contact zone is still low (Keller et al. 2020), and data on distribution and frequencies of both subspecies along the eastern and southern side of the Baltic is scarce. They present subtle plumage, song and biometric differences that overlap greatly (Hansson et al. 2000; Il'ina et al. 2021), but they can be differentiated based on slightly divergent (0.7%) and nearly fixed mitochondrial haplotypes (Helbig et al. 1996; Raković et al. 2019). In terms of migratory incidence, chiffchaffs are classified as facultative migrants (Hahn et al. 2009). A fraction of individuals spend the winter close to the breeding grounds, whereas other birds are fully migratory and perform long‐distance journeys to their wintering grounds (Lampila et al. 2009). Interestingly, ringing recoveries suggest that *collybita* birds have a more western component in their migratory routes, whereas *abietinus* seem to follow a more easterly route (Lindström et al. 2007), which is parallel to the migratory direction polymorphism displayed by *trochilus* and *acredula* willow warbler subspecies respectively (Sokolovskis et al. 2023). Hence, this motivates parallel genomic analyses in chiffchaffs with a special focus on genomic regions identified to be associated with migration direction in the willow warbler. The shared migration polymorphism between the two species could be explained by segregation of the migratory behaviour associated MARB region in both *P. trochilus* and *P. collybita* . Alternatively, the migratory polymorphism in *P. collybita* may have an entirely different genetic basis. Either way, this would provide insights into the repeatability and redundancy of complex phenotype evolution.

In this study, we investigate the common chiffchaff to compare migration and genomic differences previously characterized for its close relative, the willow warbler. First, we used light‐level geolocators to track birds from three target populations of two subspecies. This is to provide their migratory directions with a higher resolution, previously established solely by ringing recoveries (nearly absent for the subspecies *abietinus*) and to investigate individual differences. Second, we genotyped several Baltic populations of chiffchaffs for mitochondrial haplotype frequencies to locate the contact zones between the two subspecies. Together, these results helped verify that the samples selected for genome re‐sequencing are valid representatives of SW migrating pure *collybita* and SE migrating pure *abietinus*. We utilized genome re‐sequencing data to identify genomic regions divergent between the subspecies characterized by distinct migratory behaviours. In particular, we examined whether the migration‐associated regions in the willow warbler (genes or intervals in chromosomes 1, 5 and MARB) are divergent also in the chiffchaff subspecies. Finally, we estimate the time of divergence between chiffchaff subspecies by fitting demographic models to frequencies of mutation configurations in short sequence blocks (Lohse et al. 2016; Bisschop 2022; Laetsch et al. 2023). With this approach we infer an approximate ancestral effective population size and gene flow, allowing for a more accurate time of divergence than analyses involving solely summary statistics. Altogether, the results from these analyses help to predict potential hybridization between the subspecies in the two contact zones around the Baltic Sea. We are also able to study divergent regions between populations with different migratory phenotypes that might be common to willow warblers.

## 2 Methods

### 2.1 Fieldwork

We deployed 70 geolocators in chiffchaff adult males during the breeding season of 2022 across three different locations: 20 *collybita* individuals were tagged in southern Sweden (Värpinge: 55°41′36.5″ N, 13°09′56.1″ E), 30 *abietinus* individuals were tagged in northern Sweden (Tavelsjö: 64°02′08.4″ N 20°02′31.6″ E), and 20 *abietinus* individuals were further tagged in Eastern Lithuania (Mielupiai: 54°18′34.9″ N, 24°45′59.1″ E and Trakai: 54°37′00.4″ N 24°55′48.7″ E). Birds were trapped with mist nets and playback, and ringed with different combinations of metal and colour rings in order to identify them the following year. We equipped the birds with Migrate Technology Ltd. geolocators (Intigeo W30Z11‐DIP 12 × 5 × 4 mm, 0.32 g) and used the ‘leg‐loop’ harness method as in Sokolovskis et al. (2023). The logger constitutes 4% of mass relative to the average chiffchaff mass at capture (8 g). We collected 20 μL of blood from the brachial vein from each returning bird the following season (2023) for subsequent molecular analyses. The blood was stored in SET buffer (sodium chloride and sodium dodecyl sulphate) at room temperature until transferred to permanent storage at −20°C. Permits for equipping the birds with geolocators, re‐capture, and blood sampling were obtained from the relevant authorities in Sweden (Malmö‐Lund djurförsöksetiska Dnr 5.8 18‐02236/2022), Poland (resolution no. 62/2020 and 73/2021 of the Local Ethics Committee in Poznań as well as no. DZP‐WG.6401.110.2020.AS of the General Directorate for Environmental Protection), and Lithuania (Aplinkos Apsaugos Agentūra: AS‐1251).

### 2.2 Geolocator Data Analyses

The geolocator data was analysed within the R package *Geolight* (v.2.1.0). Twilight events were obtained with the ‘threshold method’ with a threshold value of 1 lx. The ‘*getElevation*’ function was used for calibration, to estimate the sun elevation angle in breeding grounds which we later used to obtain positions of the wintering grounds. The data contained a lot of noise, especially when compared to the lux values previously obtained from tracking its sister species, the willow warbler. This leads us to speculate that chiffchaffs have a more skulking behaviour and greater preference for understory. Thus, positions during the equinox periods could not be determined with certainty. Determining the wintering stationary period was similarly difficult and therefore, we selected a period (20–30 days) with the smallest standard deviation of longitude and latitude for each individual between December and March. However, one *collybita* track had to be discarded due to poor data quality, resulting in five valid tracks.

### 2.3 Mitochondrial Genotyping

We analysed DNA from 196 chiffchaffs from populations surrounding the Baltic Sea. In addition to the above‐mentioned tagging sites, these include birds sampled in south‐central Sweden, Eastern Poland and Western Poland between the years 1998 and 2022 (Table S1), the returning logger individuals (*n* = 5) and those used for resequencing (*n* = 24; see below). We extracted genomic DNA from blood or feather samples (Table S1) with the ammonium acetate protocol (Richardson et al. 2001). We diluted the DNA to 25 ng/μL and confirmed the mitochondrial haplotype by sequencing of the 5′ end of the cytochrome b gene amplified with the universal primer pair L14841 and H15149 (Kocher et al. 1989). We used GenBank reference sequences (Helbig et al. 1996) *collybita* (Z73487) and *abietinus* (Z73479), respectively, to assign the mitochondrial haplotypes. These are reported as proportions in each of six regions surrounding the Baltic Sea.

### 2.4 Reference Genome

For genomic analyses we used a high‐quality HiFi long‐read assembly from a *collybita* female (N50 = 28 Mb; Lundberg et al. 2023). The assembly used in this study was modified from the previous version in that an incorrectly assembled contig (ptg000052l) was split into a Z (ptg000518l) and W‐specific contig (ptg000052l) based on clear coverage differences between males and females. We also added a mitochondrial contig to the assembly. In this case, we first downloaded a complete mitochondrial chiffchaff sequence from GenBank (accession: OP380544.1). Next, we mapped 10× chromium reads to the assembly with the added GenBank sequence. From alignments on the mitochondrial sequence, we called variants using freebayes with default settings except for setting ploidy = 1 and filtered the raw variants using vcflib (Garrison et al. 2022). The filtered variants (*N* = 41) were added to the GenBank sequence to create a mitochondrial sequence of the focal genome sample. The updated assembly version is available at NCBI under bioproject PRJNA550489. The genome was annotated for protein‐coding genes by liftover from the southern willow warbler reference genome using liftoff version 1.6.3 (Shumate and Salzberg 2021). Repeats were annotated *de novo* for the chiffchaff genome (Lundberg et al. 2023).

Based on similarities to MARB in the willow warbler, six contigs (#32, #54, #79, #86, #107 & #117) with a total length of 28 Mb have been identified as homologous to MARB (Caballero‐Lopez et al. 2026). These are characterized by a GC content just above 50%, apparent absence of single copy genes, strong enrichment in olfactory receptors (OR) and certain repeat families, including the transposable elements diagnostic of MARB.

To provide chromosome‐scale analyses of diversity and differentiation, the chiffchaff contigs were aligned to the zebra finch genome assembly bTaeGut1_v1.p (Rhie et al. 2021) using SatsumaSynteny 2 (Grabherr et al. 2010). Contigs were assigned to specific chromosomes and ordered and oriented within them based on their longest mapping interval in the zebra finch genome. Contigs with alignments covering less than 10% of its length to any chromosome were classified as ‘unknown’. To improve the order and orientation of contigs within chromosomes, we also aligned the assembly to a highly contiguous scaffold assembly of the willow warbler (Lundberg et al. 2023) using mummer 4.0.0rc1 (Marçais et al. 2018) and placed contigs such that any telomeric sequences were either found at the start or end of the chromosome.

### 2.5 Whole‐Genome Resequencing Data

We conducted whole genome resequencing in 24 chiffchaffs (10 Swedish *abietinus*, 10 Swedish *collybita*, 2 Lithuanian *abietinus*, 2 Polish *abietinus*; Tables S1 and S2). The sequencing libraries were prepared from 1 μg DNA with a TruSeq PCR‐free sample preparation kit (cat#20015962/3, Illumina Inc.) targeting a size of 350 bp and sequenced on Novaseq 6000 S4 (Illumina) using 1.5 sequencing chemistry and a paired‐end 150 bp setup. The reads were mapped to the reference *collybita* genome using bwa mem (v0.7.17) with default parameters. The mapping was done independently for each lane. The resulting alignments were sorted using SAMtools (v.1.9; Hoff and Stanke 2019). Duplicates were then identified and marked using the Picard tool (https://broadinstitute.github.io/picard/) MarkDuplicates (GATK v4.1.4.1), and alignments from all lanes were combined.

For variant calling we used freebayes (v1.3.2; flags: ‐‐standard‐filters ‐‐min‐alternate‐count 4 ‐‐use‐best‐n‐alleles 4 ‐‐skip‐coverage 2200; Garrison and Marth 2012). Initial variant filtering was performed with vcffilter from the vcflib toolkit (v1.0.1) with the following criteria: quality score > 30, at least one alternate observation on both forward and reverse strands (SAF > 0 & SAR > 0) and balanced read support on either side of the variant (RPR > 0 & RPL > 0). We performed subsequent filtering steps with vcftools (v0.1.14; Danecek et al. 2011) where variants with low (< 10) or high (> 65) mean sequencing depth, as well as variants in repeats, were excluded to avoid low‐confidence calls and potential copy number artefacts. Each population was subsequently filtered for excessively heterozygous sites (calculated with vcftools –hardy; *p* < 2e‐3) and indels were removed. Finally, biallelic sites with missingness < 20% were kept for analyses (‐‐min‐alleles 2 ‐‐max‐alleles 2 ‐‐max‐missing 0.8 ‐‐mac2 ‐‐remove‐indels). Nucleotide diversity (π), genetic differentiation (*F*_ST_) and Tajima's *D* (*D*) were calculated using vcftools and the corresponding peaks were examined. Variant calling on the MARB contigs is challenging due to the repeat‐rich structure of this region, leading to an increased risk of mapping errors and smaller intervals where diversity could be robustly estimated. For the MARB region, we therefore refrained from within‐population diversity analyses and, for genetic differentiation, limit our analyses to the overall patterns of the region rather than focusing on the potential functional impact of specific variants or intervals.

Filtered VCF files were then converted to PLINK format using PLINK2 (v2.00‐alpha‐2.3‐20200124; Purcell et al. 2007). Variants with minor allele frequency (MAF) < 0.1 were excluded and linkage‐disequilibrium (LD) pruning was performed with PLINK (v1.90b4.9) and parameters ‐‐indep 50 5 2. PLINK was then used to run multidimensional scaling (MDS) calculations on the LD‐pruned set.

### 2.6 Demographic Analyses of Chiffchaff Subspecies

We fit demographic models of divergence with gene flow to genome sequence data from 10 *abietinus* and 10 *collybita* male individuals from Sweden. To prepare the data, raw variant calls from freebayes were filtered using gIMbleprep (Laetsch et al. 2023). This step removes indels, as well as SNPs within 2 bases of an indel, variants with QUAL < 10 and those where the alternate allele is supported exclusively by forward or reverse reads, or exclusively by the 5′ or 3′ ends of reads. Additionally, individual genotypes were set to missing if that sample had insufficient (< 8) or too high coverage (> ×2 sample‐specific mean coverage) at that site. We used gIMble to tally blockwise site frequency spectra (bSFS) from non‐repetitive intergenic regions of the genome (Laetsch et al. 2023). The length of blocks was set to 100 bases with a maximum physical span of 125 bases. The maximum count of different polymorphism types in a block (i.e., *kmax*) was set to 3, 3, 2, 2 and counts above this were recorded as > 3, > 3, > 2, > 2. We tallied a bSFS for autosomal sequences and the Z chromosome separately. To convert mutation‐scaled demographic parameters into absolute units we used the estimated mutation rate of the collared flycatcher of 4.6 × 10^−9^ mutations per site per generation for all chromosomes (Smeds et al. 2016).

We performed model fitting using agemo (Bisschop 2022) which is a python library for calculating blockwise spectra under a structured coalescent process. We fit models of Isolation with Migration where gene flow is unidirectional (testing both possible directions). We also fit a two‐epoch migration model where, backwards in time, populations never merge but are instead connected by gene flow, and effective population sizes and the rate of gene flow can change at some time in the past. Models were fit to the autosomal and Z‐specific bSFS separately. A jupyter notebook with the code for performing this analysis can be found available as a Supporting Information (MEC_SI_2).

## 3 Results

### 3.1 Distribution and Migration of Chiffchaff Subspecies

**FIGURE 1:**
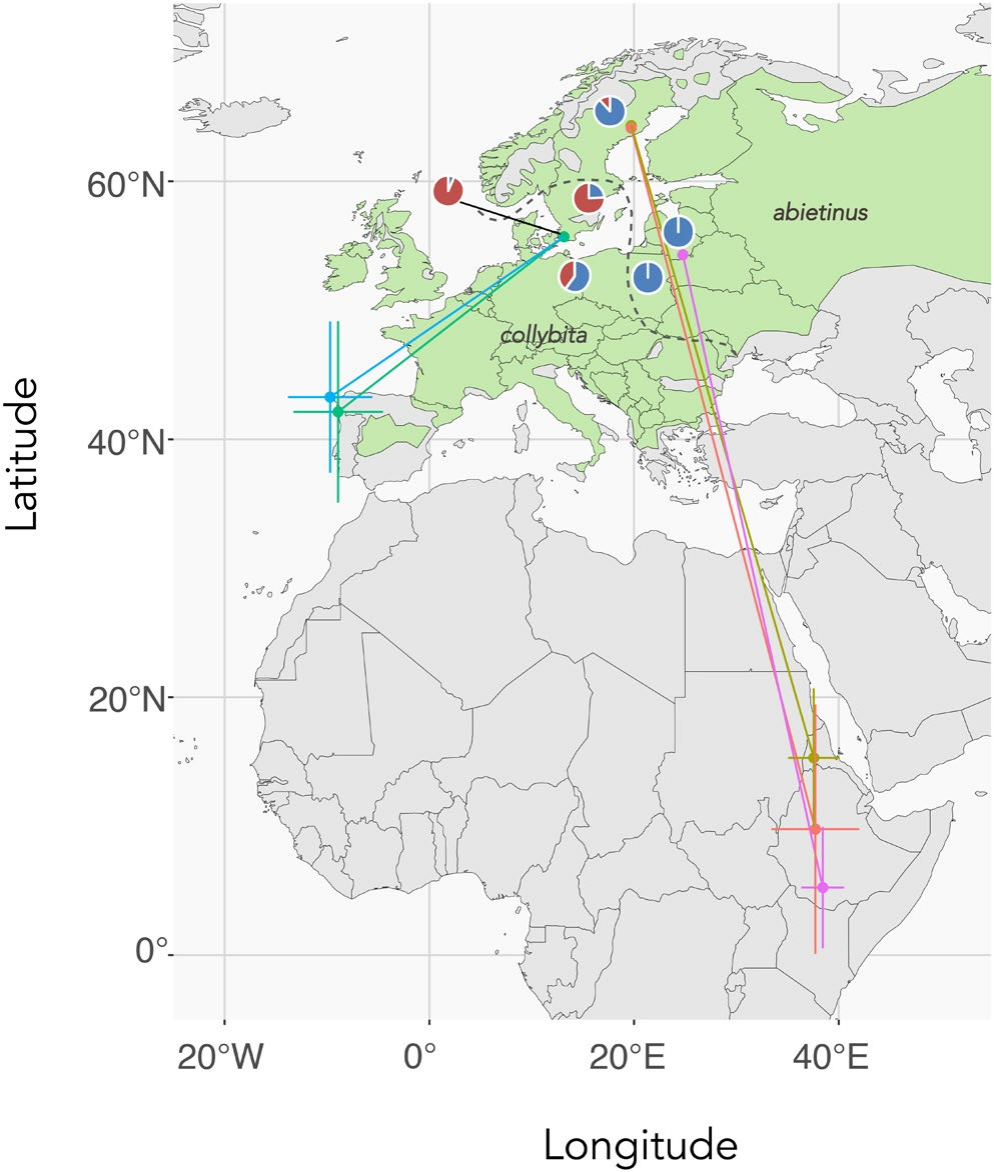
Breeding and wintering locations for each of the 5 chiffchaffs recaptured in 2023. Error bars in wintering grounds show standard deviations in longitude and latitude for each individual's wintering site. Different coloured lines connect breeding and wintering locations for each individual. Pie charts show the proportion of *abietinus* (blue) and *collybita* (red) mitochondrial haplotypes in each one of the six sampled regions around the Baltic. Sample sizes per population are: Northern Sweden, *n* = 57; South‐central Sweden, *n* = 25; Southern Sweden, *n* = 62; Western Poland, *n* = 10; Eastern Poland, *n* = 12; Lithuania, *n* = 31. The green background depicts the breeding range of *collybita* and *abietinus*, split by the contact zone (dashed line) inferred from the literature (Shirihai and Svensson 2018).

Sequencing of the partial mitochondrial cytochrome b gene shows that in Sweden, the *abietinus* haplotype is most prevalent in the north and the *collybita* haplotype dominates in the south. A minority of individuals carry a haplotype that does not match the predominant subspecies in their region (8.7% of northern birds and 4.8% of southern birds). A similar proportion of mismatches is present in phylogenetic analyses of the whole mitochondrial genome of the re‐sequenced birds (Figure S2). The sampled chiffchaff populations meet in two regions around the Baltic Sea: in South‐Central Sweden, where *collybita* and *abietinus* form a contact zone at still relatively low densities, and in western Poland, where European *collybita* encounter eastern *abietinus* birds, likely extending into Germany. The samples from Lithuania and eastern Poland are fixed for the *abietinus* haplotype (Figure 1).

Geolocator data demonstrate that chiffchaffs on either side of the contact zones follow contrasting migratory routes, establishing these zones as migratory divides. *Collybita* from Southern Sweden migrate SW and winter in the Iberian Peninsula, whereas *abietinus* from both northern Scandinavia and East of the Baltic take a SE direction crossing the Mediterranean Sea and spend the winter in East Africa (Figure 1). The migration distance is also substantially longer in *abietinus* (approx. 6000 km) than in *collybita* (approx. 2400 km). Willow warblers show the same geographic and behavioural pattern, where contact zones coincide with east–west differences in migratory direction (Sokolovskis et al. 2023).

### 3.2 Genetic Divergence Between Chiffchaff Subspecies

**FIGURE 2:**
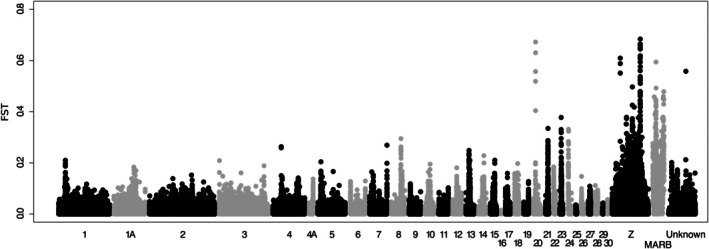
Genetic differentiation (weighted average *F*_ST_) between 14 *abietinus* and 10 *collybita* for SNPs in 10 kb windows. The windows are plotted according to their position in each contig, which in turn have been oriented, ordered and assigned to specific chromosomes based on whole‐genome alignments to the zebra finch and the willow warbler genome. Contigs that have not been assigned to a chromosome (except MARB) are presented as ‘Unknown’. *F*_ST_ estimates have only been included for windows with at least 10 SNPs and negative values have been set to 0.

**FIGURE 3:**
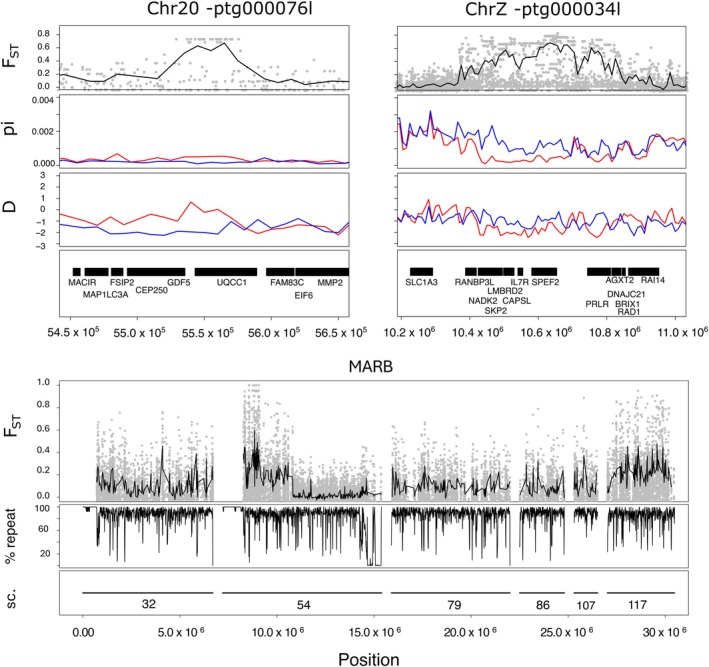
Genetic differentiation (*F*_ST_) in two intervals on chromosomes 20 (ptg000076l) and Z (ptg000034l), selected based on the longest continuous regions of high differentiation (*F*_ST_ > 0.6), and in the MARB region. *F*_ST_ has been calculated both for individual SNPs (grey) and as a weighted average in 10 kb windows. For the intervals on chromosomes 20 and Z, nucleotide diversity and Tajima's *D* have been calculated in 10 kb windows separately for collybita (red) and abietinus (blue). Windowed estimates of *F*_ST_ and Tajima's *D* have been filtered to contain a minimum of 10 SNPs. For MARB, contigs (sc.) have been ordered by their name and repeat content is displayed in 10 kb windows. Note that despite the high repeat content in the MARB region, only SNPs that do not overlap annotated repeats are included.

We explored genetic differentiation between *abietinus* and *collybita* through population summary statistics. The weighted average *F*_ST_ between 10 *collybita* and 14 *abietinus* birds for 14.56 million bi‐allelic SNPs was 0.007 (Figure 2), similar to the comparison between willow warbler subspecies (*F*_ST_ = 0.006; Lundberg et al. 2023). None of the autosomal SNPs (approximately 1.37 million) showed fixed differentiation, but SNPs with *F*_ST_ > 0.6 were concentrated on 11 chromosomes. The largest intervals of high differentiation (*F*_ST_ > 0.6) occur on chromosome 20 (contig ptg000076I) and the Z chromosome (contig ptg000034I), or on contigs assigned to MARB (Table 1; Figure 3). The regions containing the migration‐associated inversion polymorphisms in the willow warbler appear to lack highly differentiated SNPs in the chiffchaff subspecies. We provide a detailed summary of the genes overlapping variants with *F*_ST_ > 0.6 in Table S3.

**TABLE 1:** Distribution of divergent SNPs across the genomes of the chiffchaff subspecies *collybita* and *abietinus*.

Region	Average *F*_ST_	Number of SNPs			Proportion SNPs (*F*_ST_ > 0.6) × 10^−6^
		*F*_ST_ > 0.6	*F*_ST_ > 0.8	Total variants	
*Autosomes*	0.0041	60	0	13,711,465	4.37
Chr 1	0.0037	1	0	1,747,793	0.57
Chr 2	0.0039	2	0	2,245,587	0.89
Chr 3	0.0055	1	0	1,667,766	5.99
Chr 4	0.0027	12	0	1,132,973	10.59
Chr 4A	0.0034	1	0	179,859	5.56
Chr11	0.0027	2	0	293,181	6.82
Chr20	0.0035	33	0	193,843	170.24
Chr22	0.0034	1	0	65,827	15.19
Chr24	0.0051	6	0	110,075	54.51
Chr29	0.0025	1	0	31,326	31.92
Z	0.0185	590	6	618,094	954.55
*MARB* ^a^	0.0836	314	74	25,471	12327.74
*Unknown*	0.0057	8	2	179,619	44.54

^a^ MARB contigs: 32, 54, 79, 86, 107, 117.

**FIGURE 4:**
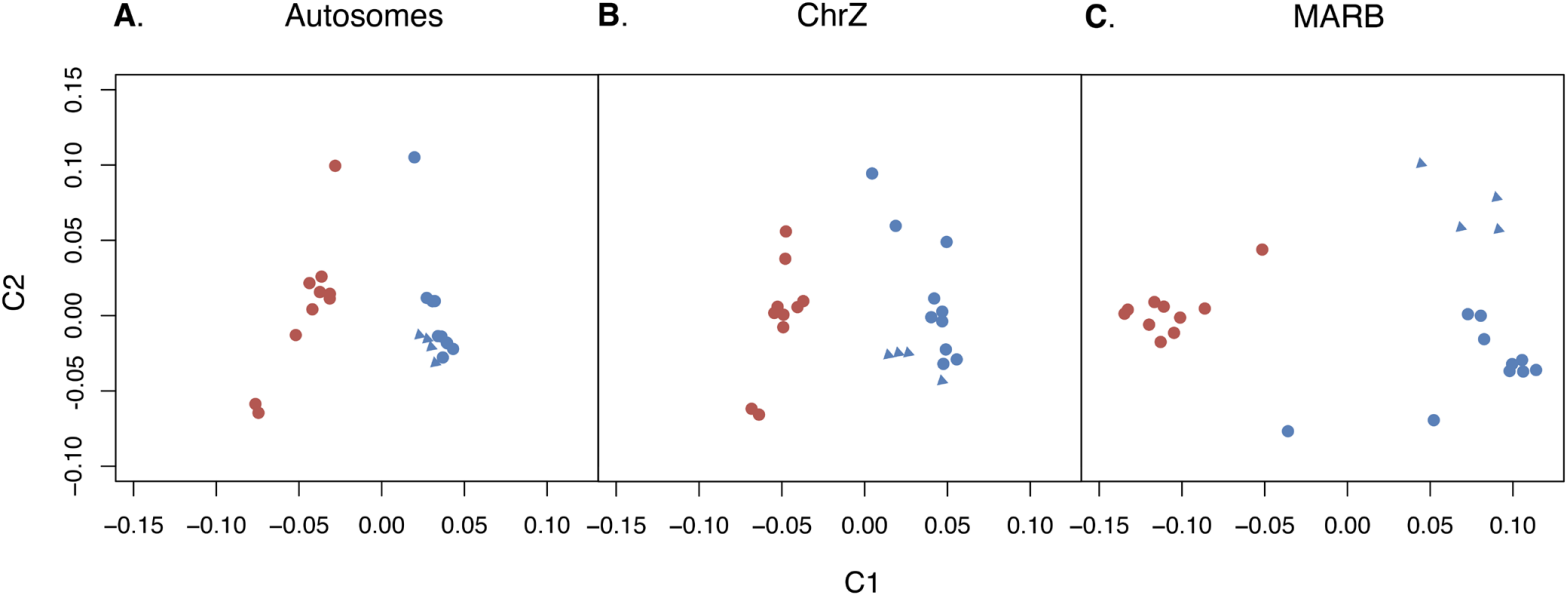
MDS plot representing genetic differentiation between Swedish *collybita* (red), Swedish *abietinus* (blue) and Polish *abietinus* (blue triangles) in (A) autosomes (1,052,539 variants), (B) Z chromosome (41,810 variants) and (C) MARB region (9094 variants).

Multidimensional scaling (MDS) plots further separate *collybita* and *abietinus* birds, irrespective of the population of origin, and such differentiation is accentuated in the region homologous to MARB (Figure 4). Nucleotide diversity was generally low for each subspecies (π ~ 0.003) and Tajima's *D* was biased towards negative values, being the lowest in *abietinus* (Autosome *D*: −1.23; Figure S3).

### 3.3 Divergence Time and Gene Flow Between Chiffchaff Subspecies

Our estimates of *F*_ST_ between the subspecies *abietinus* and *collybita* suggest that they either diverged very recently or are still connected by a high rate of gene flow. Additionally, much of the differentiation observed between the subspecies is concentrated on the Z chromosome, which could be explained by a lower effective population size (*N*_*e*_) for the Z or alternatively by stronger natural selection against gene flow on this chromosome compared to the autosomes. To investigate these questions, we fit models of divergence with gene flow to blockwise site frequency spectra (bSFS, see Methods), analyzing autosomal sequences and the Z chromosome separately.

**FIGURE 5:**
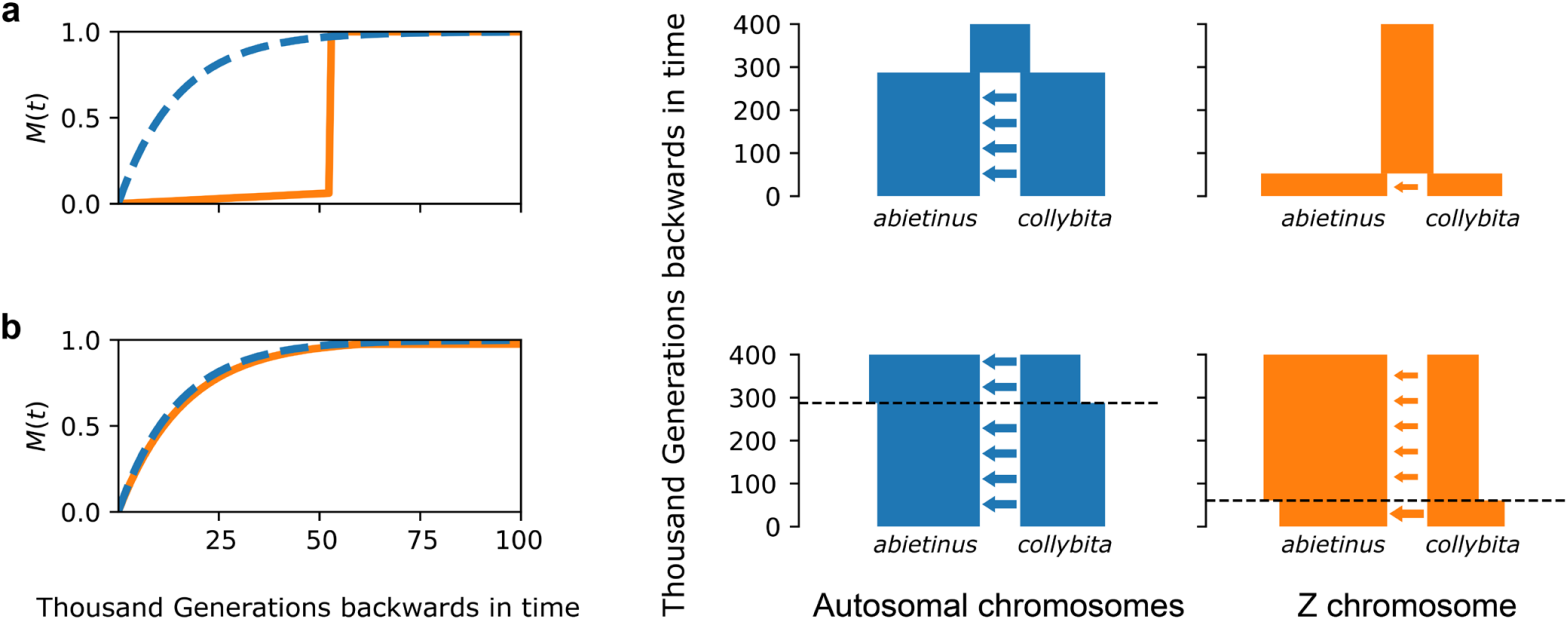
The cumulative probability distribution *M*(*t*) that *abietinus* and *collybita* lineages are in the same population at time *t*, with the dashed blue and solid orange lines corresponding to model estimates for the autosomes and Z, respectively. Estimated models of divergence with gene flow for the autosomes (blue) and Z chromosome (orange), where the width of populations is proportional to *log* (*N*_*e*_), arrows show the predominant direction of gene flow forwards in time, and the dashed line corresponds to *T*_*m*_. The top row (a) shows results for the Isolation with Migration model, and the bottom row (b) shows results for the two‐epoch Migration model, which assumes two populations connected by migration throughout time; no explicit split time is modelled int. this case (*T* → ∞).

We first fit a five‐parameter Isolation with Migration model (IM) with unidirectional gene flow (*N*_abi_, *N*_col_, *N*_anc_, *T*, *m*_*e*_; Figure 5a). This model assumes that an ancestral population splits into two at time T, with unidirectional migration connecting the two populations at rate *m*_*e*_. We find that a scenario of gene flow from *collybita* into *abietinus* (forwards in time) is favoured over gene flow in the other direction for both the autosomal chromosomes and the Z. Additionally, parameter estimates consistently suggest that contemporary populations have a larger *N*_*e*_ than the ancestral population and that *N*_*e*_ is lower overall on the Z. Estimates of the divergence time (*T*) and rate of gene flow (*m*_*e*_) do, however, differ between the autosomes and the Z. The best fitting scenario for the autosomes is one of old divergence (*T* ≈ 290,000 generations ago) with high gene flow (*m*_*e*_ ≈ 7 × 10^−5^), whereas parameter estimates for the Z suggest more recent divergence (*T* ≈ 52,000 generations ago) with a lower rate of gene flow (*m*_*e*_ ≈ 1 × 10^−6^). To better understand the difference in divergence history between the autosomes and Z we calculated the cumulative distribution for the probability that *abietinus* and *collybita* lineages are in the same population at time *t*. These distributions show that there is indeed more gene flow in recent time for the autosomes, but also that lineages are expected to be in the same population (*M*(*t*) > 0.95) by 60,000 generations in the past for both the autosomes and Z (Figure 5a).

Our estimates of gene flow through time depend on all five parameters of the Isolation with Migration model, as well as the structure of the model itself. To investigate the robustness of our results we therefore fit an alternative model of two‐epoch Migration, where populations never merge but are instead connected by gene flow and the *N*_*e*_ and *m*_*e*_ parameter can change at time *T*_m_ (Figure 5b). This model is appropriate whenever the migration rate is high enough for T to become unidentifiable: lineages will migrate and coalesce before T, erasing the signal of the split event in genome sequence data. Fitting this model to the autosomes recovers essentially the same Isolation with Migration model and *M*(*t*) as before, with only a very small improvement in likelihood (ΔlnCL = 1 × 10^−9^ per‐block). By contrast, fitting this same model to the Z leads to a higher rate of gene flow in recent time than estimated previously (*m*_*e*_ ≈ 6 × 10^−5^) and a considerable increase in likelihood (ΔlnCL = 1 × 10^−3^ per‐block). Importantly, the cumulative probabilities that lineages coalesce in the same population (*M*(*t*)) are almost identical for autosomes and Z under this model. These results therefore suggest that divergence began only recently between *abietinus* and *collybita* (~60,000 generations ago) and that there has been little difference in the rate of gene flow on the autosomes and Z.

## 4 Discussion

Here we have shown that the patterns of migratory directions of the chiffchaff subspecies *collybita* and *abietinus* occurring around the Baltic Sea significantly differ between each other. Furthermore, they mirror the directions exhibited by willow warbler subspecies (Bensch et al. 2009; Lundberg et al. 2017; Sokolovskis et al. 2023). We found that these chiffchaff subspecies have very low levels of genetic differentiation, which is mostly confined to the Z chromosome and MARB region. Interestingly, the only genomic region that is divergent between the differentially migrating subspecies in both the willow warbler and chiffchaff is MARB.

### 4.1 Migration

Most references describe wintering grounds for chiffchaffs at the species level, making subspecies‐specific distinctions difficult (Mlodinow et al. 2025). Nevertheless, detailed accounts such as Glutz von Blotzheim (1991) summarize evidence that *collybita* and *abietinus* differ in migratory routes: *collybita* typically migrates southwest towards Iberia and North Africa, whereas *abietinus* follows a more easterly route towards the Eastern Mediterranean, with occasional records extending into the Sahara and East Africa (Glutz von Blotzheim 1991; Helbig et al. 1996; Shirihai and Svensson 2018). European ringing recoveries are broadly consistent with this pattern, with only two records south of the Sahara (Uganda and Tanzania) amid predominantly Mediterranean recoveries (https://migrationatlas.org/node/1842). Our tracking data refine this picture by confirming that the two chiffchaff subspecies follow different migration routes towards their wintering grounds. *Collybita* stayed in the Iberian Peninsula and surprisingly, all three tracked *abietinus* birds reached areas in East Africa, south of the Sahara. Their wintering grounds were specifically located near the Horn of Africa, a region reported to house wintering chiffchaffs of unknown origin (Mlodinow et al. 2025). Although chiffchaffs are generally classified as facultative migrants (Hahn et al. 2009), the proportion of long‐distance migrants within *abietinus* remains unknown. The knowledge gap is particularly pronounced in the vast eastern part of the *abietinus* range, where migratory data are scarce. Recent observations nevertheless indicate overwintering along the Black Sea coast, including the Northwestern Caucasus (Dinkevich and Tilba 2022). The migratory directions followed by the tracked *collybita* and *abietinus* birds mirror those exhibited by willow warblers (Sokolovskis et al. 2023).

### 4.2 Contact Zones

Our results clarify the distribution of chiffchaff subspecies, confirm the presence of contact zones around the Baltic Sea and demonstrate divergent migratory trajectories among the interacting taxa. By genotyping 196 individuals with mtDNA markers we obtained systematic genetic confirmation of two contact zones. In South‐central Sweden, the contact is driven by the rapid northward expansion of *collybita* (Hansson et al. 2000), and it is not yet fully populated (Figure S1). Both subspecies occur together at low frequencies there, highlighting the need for continued monitoring. The other contact zone has long been depicted in the literature, with most distribution maps placing it in central Poland (Ticehurst 1938; Shirihai and Svensson 2018). However, this placement had never been confirmed by genotyping. Our data instead point to a contact zone in western Poland, probably extending into Germany, representing a shift from earlier maps. Hence, both the chiffchaff and the willow warbler show two migratory divides each, the one in Scandinavia inferred to be further south and the one in the Baltic countries inferred to be further west for chiffchaffs.

### 4.3 Genomic Divergence

Compared to the willow warbler (Lundberg et al. 2023), the overall genome diversity is lower in the chiffchaff (π = 0.003 versus π = 0.006). This contrasts with the mitochondrial genome, which is virtually undifferentiated in the willow warbler but shows greater diversity and nearly fixed differences between chiffchaff subspecies (Bensch et al. 2006; Raković et al. 2019).

Although mtDNA divergence between *collybita* and *abietinus* is pronounced, the small population size of the mitochondria means that a higher degree of differentiation compared to the autosomes is expected under neutrality (Moore 1995). Consistent with this, no fixed SNPs were detected in the nuclear genome (Table 1), yet the subspecies can be confidently separated by MDS analyses based on filtered SNPs, both for the autosomes and the Z chromosome (Figure 3). This suggests that this divergence is driven by shifts in allele frequencies across many loci. This pattern contrasts with the willow warbler where the two subspecies cannot be separated outside the regions affected by the inversion polymorphisms (Lundberg et al. 2017) or MARB (Caballero‐López et al. 2022; Sokolovskis et al. 2023). Notably, the corresponding regions in the chiffchaff genome (on chromosomes 1, 3 and 5) do not contain SNPs or windows with exceptionally high *F*_ST_ values. Instead, the highest concentration of highly differentiated SNPs (*F*_ST_ > 0.6) in the contigs mapped to autosomes occurs in a region of chromosome 20 containing the *UQCC1* gene (Figure 3b). This gene is involved in mitochondrial complex III assembly and cytochrome b stability (Tucker et al. 2013). Given the differentiated mitochondrial haplotypes in chiffchaffs, specifically in the cytochrome b gene, this leads us to speculate whether the divergence in nuclear genes related to mitochondrial function (mitonuclear coevolution; Morales et al. 2018) may contribute to incipient subspecies differentiation in the chiffchaff.

The accumulation of *F*_ST_ peaks is notably greater in the Z chromosome, meanwhile π is reduced. The observed divergence and lower diversity could well be a consequence of the Z chromosome's smaller *N*_*e*_, which makes the Z chromosome prone to the faster accumulation of germline ‐and sometimes mildly deleterious‐ mutations while experiencing stronger genetic drift (Ellegren 2007; Mank et al. 2010). Because of this, elevated *F*_ST_ values on the Z chromosome are not uncommon in birds (Oyler‐McCance et al. 2015). However, the largest cluster of highly differentiated SNPs on the Z chromosome is located in an interval that includes the *SPEF2* gene, which is involved in sperm flagella development across several taxa (Sironen et al. 2011; Li et al. 2022). Interestingly, *SPEF2* has been previously associated with *PRLR* (Elferink et al. 2008), a gene that is also included in the same differentiated block. *PRLR* is involved in the induction of moulting (Juhn and Harris 1958) and egg production (Cui et al. 2006). As these genes influence reproductive traits, the observed differentiation on the Z chromosome may contribute to emerging reproductive incompatibilities between *abietinus* and *collybita* (Sætre et al. 2003). Z‐linked patterns of speciation have been widely observed across bird taxa such as *Ficedula* flycatchers in the Palearctic (Ellegren et al. 2012) or Common Yellowthroats (*G. t. trichans*) in the Nearctic (Dunn et al. 2024). However, in comparison, *abietinus* and *collybita* seem to be at an early stage of divergence.

The accumulation of highly differentiated SNPs (*F*_ST_ > 0.8, 11 times higher than the genomic average) in contigs homologous to the MARB region in the willow warbler suggests that this genomic block may also play a role in subspecies divergence in chiffchaffs. An increased Tajima's *D* and a reduced nucleotide diversity in both subspecies point towards population structure (Figure S3) or potentially reduced gene flow in this region. These patterns may reflect sequence variation but also structural differences as mapping on this complex region is challenging (variation in coverage and collapsed repeats). While this limits our ability to assess within‐population diversity and selection directly, the consistent differentiation between subspecies implies that MARB may be implicated in the early stage of divergence, possibly through an effect on migratory routes as shown in willow warblers (Sokolovskis et al. 2023). MARB contains a high density of OR genes and repeats such as transposable elements (Caballero‐López et al. 2022) and so it has been suggested that any effect on the migratory phenotype is likely regulatory (Caballero‐Lopez and Bensch 2024). Complex repeat‐rich regions of this kind are often involved in epigenetic processes including chromatin conformation, DNA packaging and methylation or non‐coding RNA, which affect gene expression (Huda et al. 2009; Yandım and Karakülah 2019). Importantly, these mechanisms often affect neural circuit wiring during early development where many behaviours are determined (Soutschek and Schratt 2023). It is thus exciting to speculate on a scenario where differently migrating subspecies would interpret magnetic or spatial information differently due to their divergent MARB contributing to distinct migratory routes. In addition, copy number variation in transposable elements and other repeats is known to contribute to structural variation between closely related taxa (Poignet et al. 2021) and has been linked to the diversification of passerine birds into thousands of species (Suh et al. 2018). Taken together, these features make MARB a plausible candidate for influencing migratory behaviour through regulatory or structural mechanisms, even though its precise role remains to be determined.

### 4.4 Demographic Analyses and Divergence Times

Our demographic modelling results suggest that the chiffchaff subspecies diverged recently, around 100,000 years ago (~60,000 generations), assuming a generation time of 1.7 years (Bensch et al. 1999). This is broadly consistent with previous estimates of divergence based on mtDNA (~150,000 years ago or 80,000 generations; Raković et al. 2019). Our results also indicate a high rate of historical gene flow predominantly from *collybita* to *abietinus*, which likely contributed to overall low genetic differentiation between the subspecies.

A plausible scenario resulting from the best‐fitting model (two‐epoch Migration) is that ancestral chiffchaff populations diverged during the last glaciation (115,000–11,700 years ago; Clement and Peterson 2008; Hansson et al. 2000) despite the substantial gene flow. This divergence might have been facilitated by lower *N*_*e*_ at the time, which is not uncommon during glaciation maxima (Hewitt 1996; Thorup et al. 2021).

### 4.5 Hybridisation

Our analyses focused on individuals from allopatric populations of *collybita* and *abietinus*, in order to better detect divergent regions. This design likely explains why we did not encounter any apparent hybrid individuals among the resequence samples (Figure 4). However, the overall low differentiation between *collybita* and *abietinus*, together with the mtDNA introgression in both subspecies (Figure S2) and their recent divergence times suggest that hybridisation is happening in the contact zones. This is especially likely in the Polish divide, which is older and contains higher densities (Keller et al. 2020). Frequent hybridisation has been documented between chiffchaff taxa that are more genetically and phenotypically divergent than *abietinus* and *collybita*. For instance, Iberian chiffchaffs ( *P. ibericus* ) often interbreed with *collybita* in a narrow area of the western Pyrenees (Bensch, Helbig, et al. 2002; Helbig et al. 2001). Likewise, there is a hybrid zone between the *abietinus* and *tristis* subspecies, despite phenotypic differences in morphology and song characteristics (Marova et al. 2009). Though mtDNA and nuclear genome differentiation is slightly greater in these two subspecies, genetic admixture is still common between them (Shipilina et al. 2017). These cases support the idea that the relatively closer genetic relationship between *abietinus* and *collybita* likely facilitates hybridisation in the contact zones.

## 5 Conclusion

The two investigated chiffchaff subspecies follow distinct migratory routes (both in direction and distance) to their wintering grounds. Furthermore, they meet in two contact zones of different age: a recent one in south‐central Scandinavia and an older one in Western Poland, where hybridisation is very likely taking place. Further resequencing of individuals from the contact zones will help quantify the extent of hybridisation between both subspecies. Genome resequencing data show that the differentiation patterns between *abietinus* and *collybita* correspond to taxa in early stages of divergence. This is supported by demographic modelling, which estimated such divergence to begin around 100,000 years ago. Still, birds from each subspecies can be confidently assigned to their respective populations, suggesting that differences are likely spread through many loci. Whether the divergence is a result of selection or drift due to reduced historical *N*_*e*_ remains hard to say. The strongest amount of differentiation in the chiffchaff genome happens in the MARB region, which segregates SE and SW migrating willow warblers.

It is important to note that due to the highly repetitive nature of MARB, it is a very challenging region to resolve using short‐read NGS data. Mapping and variant calling can lead to biased interpretation of genetic variation in this region, particularly regarding structural variation and haplotype detection. Consequently, both the extent of genetic variation and structure of MARB should be studied with caution. In particular, we do not know in detail how MARB haplotypes differ between the chiffchaffs and willow warblers, and to what extent they share an evolutionary history or reflect convergent evolution. To reliably compare the region across the two species and also between subspecies would require even more contiguous genome assemblies, ideally where the MARB chromosome is assembled from telomere to telomere. The exact mechanism behind MARB's influence on migratory routes is still unknown, and future analyses involving expression studies will help elucidate it. The presence of this region across several (and possibly all) *Phylloscopus* (Caballero‐Lopez et al. 2026) suggests that its effect on migration could transcend species boundaries.

## Author Contributions

Conceptualization: V.C.‐L., S.B. Field data collection: V.C.‐L., S.B., G.M., M.P., Ł.J., M.B. Laboratory work: V.C.‐L., M.B. Tracking data analysis: V.C.‐L. Bioinformatic analyses: A.M., D.E., E.P.‐W., M.L. Writing: V.C.‐L. Review and editing: all co‐authors.

## Funding

This work was supported by Vetenskapsrådet (2021‐03853), Erik Philp‐Sörensens Foundation (G2022‐014), and Kungliga Fysiografiska Sällskapet i Lund.

## Conflicts of Interest

The authors declare no conflicts of interest.

## Supporting information


**Figure S1:** Range expansion of chiffchaffs in Sweden (1996–2022). Swedish Bird Survey, unpublished.
**Figure S2:** Maximum likelihood tree of the whole mitochondrial genome from the 26 genome‐resequenced chiffchaffs. The tree is rooted with two willow warblers (*P. t. trochilus* in medium grey, *P. t. acredula* in light grey) and a dusky warbler ( *P. fuscatus* , dark grey) from Lundberg et al. (2023). The colour of the sample names indicate subspecies based on catching location; blue—*abietinus*, red—*collybita*. The tree was constructed in MEGA 12.0 using the General Time Reversible model + G (0.336) + I (47.6%). Scale bar = 0.01 substitutions per site.
**Figure S3:** Density plots of *F*_ST_, nucleotide diversity and Tajima's *D* in 10 kb windows for Z‐linked contigs, MARB‐associated contigs and the rest of the genome (Autosomes). *F*_ST_ and Tajima's *D* have been filtered to contain only windows with at least 10 SNPs.
**Table S1:** Summary of all chiffchaffs used for the present study (196 individuals). It includes feather samples for genotyping (green), blood samples for resequencing and genotyping (yellow), blood samples from logger birds and genotyping (blue), and blood for only genotyping (no colour).
**Table S2:** Detailed sequencing procedure of all chiffchaff individuals used for population analyses and MDS. The read depth measurements correspond to the mean for each sample in the filtered vcf file.
**Table S3:** Genes overlapping SNPs with *F*_ST_ > 0.6 in contigs assigned to chromosomes. Contigs that could not be assigned to a specific chromosome did not render high *F*_ST_ SNPs overlapping annotated genes. MARB does not contain any single copy gene and the assignment of variants to specific gene copies is difficult.

## Data Availability

Data that supports the findings of this study are mostly available in the Supporting Information of this article. All resequence data is available at ENA (European Nucleotide Archive) under project PRJEB115107.
