# Supplementary material for: Common Chiffchaffs (Phylloscopus collybita) Diverge in a Genomic Region Associated With Migration Differences in Willow Warblers (Phylloscopus trochilus)
Source: Mol Ecol. 2026 Jul 9;35(13):e70462. doi: 10.1111/mec.70462 (PMC13347281; doi:10.1111/mec.70462)
Supplement: Supplementary file 1 — Figure S1: Range expansion of chiffchaffs in Sweden (1996–2022). Swedish Bird Survey, unpublished. Figure S2: Maximum likelihood tree of the whole mitochondrial genome from the 26 genome‐resequenced chiffchaffs. The tree is rooted with two willow warblers (P. t. trochilus in medium grey, P. t. acredula in light grey) and a dusky warbler ( P. fuscatus , dark grey) from Lundberg et al. (2023). The colour of the sample names indicate subspecies based on catching location; blue—abietinus, red—collybita. The tree was constructed in MEGA 12.0 using the General Time Reversible model + G (0.336) + I (47.6%). Scale bar = 0.01 substitutions per site. Figure S3: Density plots of F ST, nucleotide diversity and Tajima's D in 10 kb windows for Z‐linked contigs, MARB‐associated contigs and the rest of the genome (Autosomes). F ST and Tajima's D have been filtered to contain only windows with at least 10 SNPs. Table S1: Summary of all chiffchaffs used for the present study (196 individuals). It includes feather samples for genotyping (green), blood samples for resequencing and genotyping (yellow), blood samples from logger birds and genotyping (blue), and blood for only genotyping (no colour). Table S2: Detailed sequencing procedure of all chiffchaff individuals used for population analyses and MDS. The read depth measurements correspond to the mean for each sample in the filtered vcf file. Table S3: Genes overlapping SNPs with F ST > 0.6 in contigs assigned to chromosomes. Contigs that could not be assigned to a specific chromosome did not render high F ST SNPs overlapping annotated genes. MARB does not contain any single copy gene and the assignment of variants to specific gene copies is difficult. [file MEC-35-e70462-s001.docx]

**Supplemental Information for:**

**Common chiffchaffs (*Phylloscopus collybita*) diverge in a genomic region associated to migration differences in willow warblers (*Phylloscopus trochilus*).**

Violeta Caballero-Lopez^1^, Alexander Mackintosh^2^, Diana Ekman^3^, Estelle Proux-Wéra^3^, Max Lundberg^1^, Gintaras Malmiga^4^, Daria Shipilina^5^, Michal Polakowski^6^, Michaëla Berdougo^1^, Łukasz Jankowiak^6^, Staffan Bensch^1^.

**Table of Contents:**

| **Figure S1** | Page 2 |
| --- | --- |
| **Figure S2** | Page 3 |
| **Figure S3** | Page 4 |
| **Table S1** | Page 5-9 |
| **Table S2** | Page 9 |
| **Table S3** | Page 10-11 |


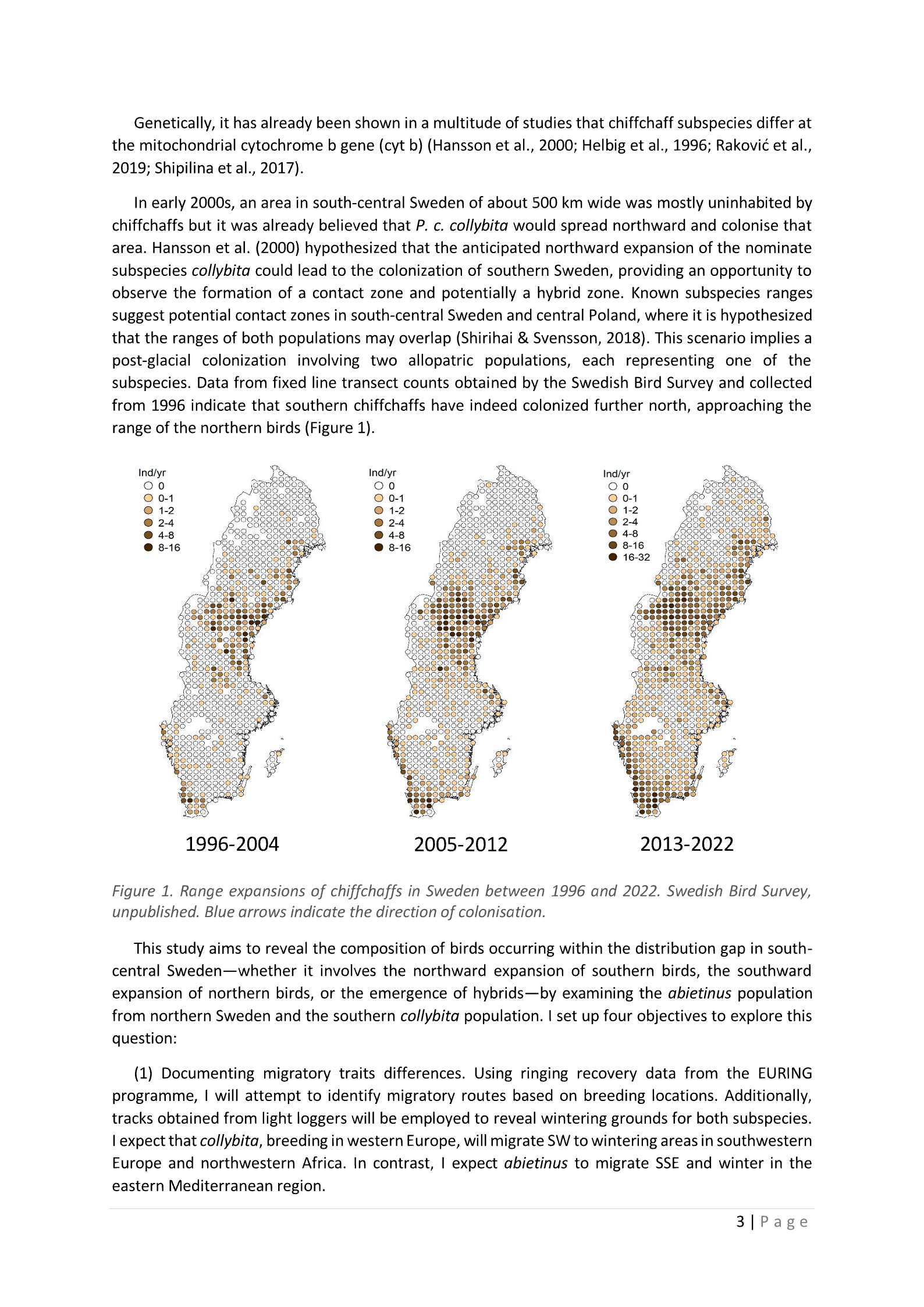


**Figure S1.** Range expansion of chiffchaffs in Sweden (1996-2022). Swedish Bird Survey, unpublished

**Figure S2.** Maximum likelihood tree of the whole mitochondrial genome from the 26 genome-resequenced chiffchaffs. The tree is rooted with two willow warblers (*P. t .trochilus* in medium grey, *P. t. acredula* in light grey) and a dusky warbler (*P. fuscatus*, dark grey) from Lundberg et al. (2023). The colour of the sample names indicate subspecies based on catching location; blue - *abietinus*, red – *collybita*. The tree was constructed in MEGA 12.0 using the General Time Reversible model + G (0.336) + I (47.6%). Scale bar = 0.01 substitutions per site.

**Figure S3**. Density plots of FST, nucleotide diversity and Tajima’s D in 10 kb windows for Z-linked contigs, MARB-associated contigs and the rest of the genome (Autosomes). F_ST_ and Tajima’s D have been filtered to contain only windows with at least 10 SNPs.

**Table S1.** Summary of all chiffchaffs used for the present study (196 individuals). It includes feather samples for genotyping (green), blood samples for resequencing and genotyping (yellow), blood samples from logger birds and genotyping (blue), and blood for only genotyping (no color).

**Table S2**. Detailed sequencing procedure of all chiffchaff individuals used for population analyses and MDS. The read depth measurements correspond to the mean for each sample in the filtered vcf file.

| Population | Number of individuals | Read depth (mean) | Read depth (Range) |
| --- | --- | --- | --- |
| Sweden *Abietinus* | 10 | 35.45 X | 26.83 – 51.44 |
| Sweden *Collybita* | 10 | 32.35 X | 24.66 – 41.82 |
| Poland *Abietinus* | 2 | 37.8 X | 34.00 – 41.61 |
| Lithuania *Abietinus* | 2 | 34.28 X | 33.89 – 34.67 |

**Table S3.** Genes overlapping SNPs with F_ST_ > 0.6 in contigs assigned to chromosomes. Contigs that could not be assigned to a specific chromosome did not render high F_ST_ SNPs overlapping annotated genes. MARB does not contain any single copy gene and the assignment of variants to specific gene copies is difficult.

| Chromosome | Gene | Name | Function in humans |
| --- | --- | --- | --- |
| 1 | *FAT3* | FAT atypical cadherin 3 | Neuronal development and cell-cell adhesion. Associated with spinocerebellar ataxia. |
| 2 | *CROT* | carnitine O-octanoyltransferase | Involved in lipid metabolism, beta-oxidation in peroxisomes. |
| 4 | *ANK2* | ANKYRIN2 | Cell motility, activation, proliferation, contact and the maintenance of specialized membrane domains. Linked to cardiac arrhythmias. |
|  | *FGFR3* | Fibroblast growth factor receptor 3 | A receptor tyrosine kinase invoilved in bone growth and cell signalling. Mutations associate skeletal dysplasias. |
| 11 | *HSBP1* | heat shock factor binding protein 1 | Regulates heat shock response by interacting with HSF1. Involved in cell stress tolerance. |
| 20 | *GDF5* | growth differentiation factor | Growth factor crucial for cartilage and bone formation, linked to osteoarthritis and skeletal disorders. |
|  | *UQCC1* | ubiquinol-cytochrome c reductase complex assembly factor 1 | mitochondrial complex III assembly and cytochrome b stability. Polymorphisms are associated with variation in height and osteoarthritis. |
| 24 | *TEK* | Receptor tyrosine kinase | Encodes Tie2 receptor, essential for vascular development and angiogenesis. Mutations in this gene are associated with inherited venous malformations of the skin and mucous membranes. |
| 29 | *ITGA7* | Integrin subunit Alpha 7 | Encodes integrin alpha-7, a laminin receptor in skeletal and cardiac muscle. |
| Z | ZFYVE16 | zink finger FYVE-type containing 16 | Encodes FYVE-type zinc finger protein involved in endosomal trafficking and TGF-beta signalling. Plays a role in cell growth and differentiation. |
|  | NPR2 | Natriuretic peptide receptor 2 | Guanylate cyclase that produces cGMP; involved in skeletal development and growth regulation. |
|  | PRLR | Prolactin Receptor | Regulates broodiness, crop milk production; candidate gene for poultry breeding.* |
|  | NTRK2 | Neurotrophic Receptor tyrosine kinase 2 | Receptor for BDNF; critical for neuronal survival, development, and synaptic plasticity. |
|  | MLLT3 | Myeloid/Lymphoid or Mixed-Lineage Leukemia; Translocated to, 3 | Transcriptional regulator; involved in hematopoiesis . |
|  | *QRICH2* | Glutamine Rich 2 | Involved in sperm flagellar structure and motility; linked to male fertility. |
|  | *RANBP3L* | RAN Binding Protein 3 Like | May be involved in nuclear transport of proteins, bone development. |
|  | *NADK2* | NAD Kinase 2 | Mitochondrial enzyme converting NAD to NADP; essential for redox balance. |
|  | *LMBRD2* | LMBR1 Domain Containing 2 | Involved in adrenergic receptor signaling pathway. |
|  | *CAPSL* | Calcyphosine-Like | Calcium-binding protein; may play a role in signal transduction. |
|  | *IL7R* | Interleukin 7 Receptor | Key receptor in T-cell development and immune regulation. Lymphocyte development. |
|  | *SPEF2* | Sperm Flagellar 2 | Essential for sperm motility and flagellar structure.* |
|  | *CCDC125* | Coiled-Coil Domain Containing 125 | Involved in activation of GTPase activity; negative regulation of Rho protein signal transduction; and negative regulation of cell motility |
